# Supplementary material for: Phagocytosis is mediated by two-dimensional assemblies of the F-BAR protein GAS7
Source: Nat Commun. 2019 Oct 18;10:4763. doi: 10.1038/s41467-019-12738-w (PMC6802115; doi:10.1038/s41467-019-12738-w)
Supplement: Supplementary file 1 — Supplementary Information [file 41467_2019_12738_MOESM1_ESM.pdf]

Supplementary Information

Phagocytosis is mediated by two-dimensional assemblies of the F-BAR protein GAS7

Kyoko Hanawa-Suetsugu , Yuzuru Itoh, Maisarah Ab Fatah, Tamako Nishimura et al.

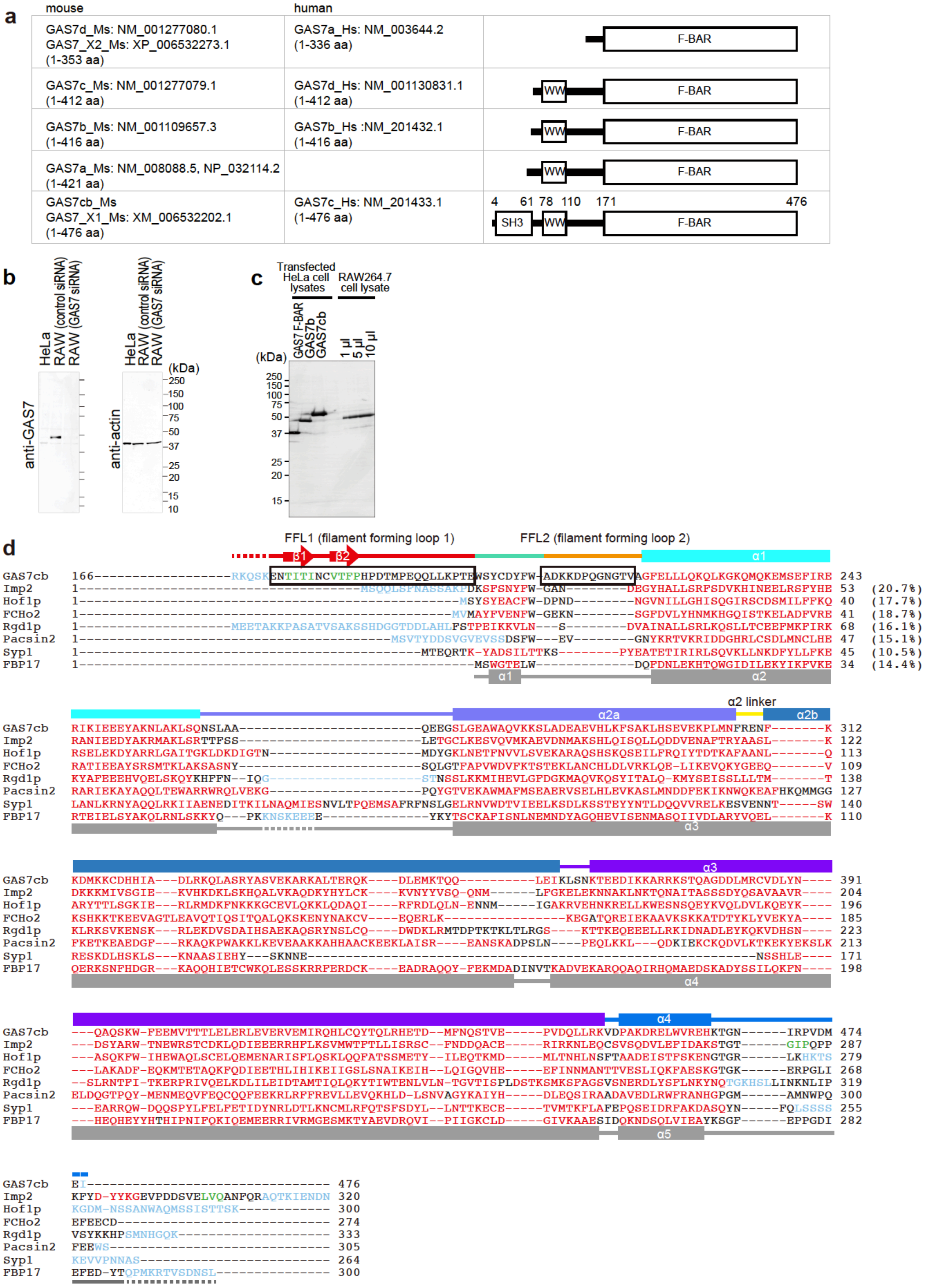

a
mouse
human
GAS7d_Ms: NM_001277080.1
GAS7_X2_Ms: XP_006532273.1
(1-353 aa)
GAS7a_Hs: NM_003644.2
(1-336 aa)
F-BAR
GAS7c_Ms: NM_001277079.1
(1-412 aa)
GAS7d_Hs: NM_001130831.1
(1-412 aa)
WW
F-BAR
GAS7b_Ms: NM_001109657.3
(1-416 aa)
GAS7b_Hs :NM_201432.1
(1-416 aa)
WW
F-BAR
GAS7a_Ms: NM_008088.5, NP_032114.2
(1-421 aa)
WW
F-BAR
GAS7cb_Ms
GAS7_X1_Ms: XM_006532202.1
(1-476 aa)
GAS7c_Hs: NM_201433.1
(1-476 aa)
4 61 78 110 171 476
SH3
WW
F-BAR
b
HeLa
RAW (control siRNA)
RAW (GAS7 siRNA)
anti-GAS7
anti-actin
(kDa)
250
150
100
75
50
37
25
20
15
10
c
Transfected HeLa cell lysates
RAW264.7 cell lysate
GAS7 F-BAR
GAS7b
GAS7cb
1 µl
5 µl
10 µl
(kDa)
250
150
100
75
50
37
25
20
15
d
FFL1 (filament forming loop 1)
FFL2 (filament forming loop 2)
β1
β2
α1
GAS7cb 166------------------RKQSKENTITINCVTFPHPDTMPEQQLLKPTEWSYCDYFW-ADKKDPQGNGTVAGFELLLQKQLKGKQMQKEMSEFIRE 243
Imp2 1------------------------------MSQQLSFNASSAKPDKSFSNYFW-GAN--------DEGYHALLSRFSDVKHINEELRSFYHE 53 (20.7%)
Hof1p 1---MSYSYEACFW-DPND--------NGVNILLGHISQGIRSCDSMILFFKQ 40 (17.7%)
FCHo2 1---MVMAYFVENFW-GEKN--------SGFDVLYHNMKHGQISTKELADFVRE 41 (18.7%)
Rgd1p 1------------------MEETAKKPASATVSAKSSHDGGTDDLAHLFSTPEIKKVLN---S--------DVAINALLSRLKQSLLTCEEFMKFIRK 68 (16.1%)
Pacsin2 1-------------------------------------MSVTYDDSVGVEVSSDSFW---EV--------GNYKRTVKRIDDGHRLCSDLMNCLHE 47 (15.1%)
Sypl 1---MTEQRTK-YADSILTTKS--------PYEATETIRIRLSQVKLLNKDFYLLFKE 45 (10.5%)
FBP17 1---MSWGTELW--------------DQFDNLEKHTQWGIDILEKYIKFVKE 34 (14.4%)
α1
α2
α2 linker
α2a
α2b
GAS7cb RIKIEEEYAKNLAKLSQNSLAA-----------------------QEEGSLGEAWAQVKKSLADEAEVHLKFSAKLHSEVEKPLMNFRENF-------K 312
Imp2 RANIEEDYAKRMAKLSRTTFSS-----------------------LETGCLKESVQVMKAEVDNMAKSHLQISQLLQDDVENAFTRYAASL-------K 122
Hof1p RSELEKDYARRLGAITGKLDKDIGTN-------------------MDYGKLNETFNVVLSVEKARAQSHSKQSEILFRQIYTDTKAFAANL-------Q 113
FCHo2 RATIEEAYSRSMTKLAKSASNY-----------------------SQLGTFAPVWDVFKTSTEKLANCHLDLVRKLQE-LIKEVQKYGEEQ-------V 109
Rgd1p KYAFEEEHVQELSKQYKHFFN--IQG-------------------STNSSLKKMIHEVLGFDGKMAQVKQSYITALQ-KMYSEISSLLLTM-------T 138
Pacsin2 RARIEKAYAQQLTEWARRWRQLVEKG-------------------PQYGTVEKAWMAFMSEAERVSELHLEVKASLMNDDFEKIKNWQKEAFHKQMMGG 127
Sypl LANLKRNYAQQLRKIIAENEDITKILNAQMIESNVLTPQEMSAFRFNSLGELRNVWDTVIEELKSDLKSSTEYYNTLDQQVVRELKESVENNT-----SW 140
FBP17 RTEIELSYAKQLRNLSKKYQ---PKKNSKEEEE-------------YKYTSCKAFISNLNEMNDYAGQHEVISENMASQIIVDLARYVQEL-------K 110
α3
α3
GAS7cb KDMKKCDHHIA---DLRKQLASRYASVEKARKALTERQK----DLEMKTQQ----------LEIKLSNKTEEDIKKARRKSTQAGDDLMRCVDLYN---- 391
Imp2 DKKKMIVSGIE---KVHKDKLSKHQALVKAQDKYHYLCK----KVNYYVSQ-QNM------LFGKELEKNNAKLNKTQNAITASSSDYQSAVAAVR---- 204
Hof1p ARYTTLSGKIE---RLRMDKFNKKKGCEVLQKKLQDAQI----RFRDLQLN-ENNM-----IGAKRVEHNKRELLKWESNSQEYKVQLDVLKQEYK---- 196
FCHo2 KSHKKTKEEVAGTLEAVQTIQSITQALQKSKENYNAKCV----EQERLK-----------------KEGATQREIEKAAVKSKKATDTYKLYVEKYA---- 185
Rgd1p KLRKSVKENSK---RLEKDVSDAIHSAEKAQSRYNSLCQ----DWDKLRMTDPTKTKLTLRGS----KTTKEQEEELLRKIDNADLEYKQKVDHSN---- 223
Pacsin2 FKETKEAEDGF---RKAQKPWAKKLKEVEAAKKAHHAACKEEKLAISR----EANSKADPSLN----PEQLKKL---QDKIEKCKQDVLKTKEKYEKSLK 213
Sypl RESKDLHSKLS---KNAASIEHY---SKNNE---NSSHLE---- 171
FBP17 QERKSNFHDGR---KAQQHIETCWKQLESSKRRFERDCK----EADRAQQY-FEKMDADINVTKADVEKARQQAQIRHQMAEDSKADYSSILQKFN---- 198
α4
α4
α5
GAS7cb ---QAQSKW-FEEMVTTTLELERLEVERVEMIRQHLCQYTQLRHETD--MFNQSTVE-----PVDQLLRKVDPAKDRELWVREHKTGN------IRPVDM 474
Imp2 ---DSYARW-TNEWRSTCDKLQDIEEERRHFLKSVMWTFTLLISRSC--FNDDQACE-----RIRKNLEQCSVSQDVLEFIDAKSTGT------GIPQPP 287
Hof1p ---ASQKFW-IHEWAQLSCELQEMENARISFLQSKLQQFATSSMETY--ILQTKMD------MLTNHLNSFTAADEISTFSKENGTGR------LKHKTS 279
FCHo2 ---LAKADF-EQKMTETAQKFQDIEETHLIHIKEIIGSLSNAIKEIH--LQIGQVHE-----EFINNMANTTVESLIQKFAESKGTGK------ERPGLI 268
Rgd1p ---SLRNTFI-TKERPRIVQELKDLILEIDTAMTIQLQKYTIWTENLVLN-TGVTISPLDSTKSMKSFAGSVSNERDLYSFLNKYNQTGKHSLLINKNLIP 319
Pacsin2 ELDQGTPQY-MENMEQVFEQCQQFEEKRLRFFREVLLEVQKHLD-LSNVAGYKAIYH-----DLEQSIRAADAVEDLRWFRANHGPGM------AMNWPQ 300
Sypl ---EARRQW-DQQSPYLFELFETIDYNRLDTLKNCMLRFQTSFSDYL--LNTTKECE-----TVMTKFLAFEPQSEIDRFAKDASQYN------FQLSSSS 255
FBP17 ---HEQHEYYHTHIPNIFQKIQEMEERRIVRMGESMKTYAEVDRQVI--PIIGKCLD-----GIVKAAESIDQKNDSQLVIEAYKSGF------EPPGDI 282
GAS7cb EI------------------------------ 476
Imp2 KFYD-YYKGEVPDDSVELVQANFQRAQTKIENDN 320
Hof1p KGDM-NSSANWAQMSSISTTSK------------ 300
FCHo2 EFEECD---------------------------- 274
Rgd1p VSYKKHPSMNHGQK-------------------- 333
Pacsin2 FEEWS----------------------------- 305
Sypl KEVVPNNAS------------------------- 264
FBP17 EFED-YTQPMKRTVSDNSL--------------- 300

Supplementary Figure 1. GAS7 splicing isoforms and structure-based alignment of the F-BAR domain sequences.

(**a**) Mouse (*Mus musculus*) and human (*Homo sapiens*) splicing isoforms of GAS7, with their domain structures and GenBank accession numbers. The amino acid residue numbers of GAS7cb are indicated.

(**b**) Western blot showing GAS7 expression in HeLa cells and RAW264.7 macrophage cells treated with control or GAS7 siRNAs. Actin is shown as a control. HeLa cells did not display detectable GAS7.

(**c**) GAS7 isoforms expressed in RAW264.7 cells. Increasing numbers of non-transfected RAW264.7 cells were analysed by western blotting, together with HeLa cells transfected with plasmids encoding the GAS7 F-BAR fragment (similar molecular weight to human GAS7a or mouse GAS7d), GAS7b, or GAS7cb, using an anti-GAS7 antibody.

(**d**) Amino-acid sequences of F-BAR domain proteins, aligned based on their three-dimensional structures. Secondary structure elements of the F-BAR domains of GAS7 and FBP17 are shown above and below the alignment, respectively. Secondary structure elements of the GAS7 F-BAR domain are coloured as in Figure 1a. The GAS7-specific FFL1 and FFL2 and the α2 linker are indicated. Residues are coloured based on the corresponding secondary structures determined from the crystal structures: red, α helices; green, β sheets; cyan, disordered region. The percentages of amino acid sequence identity between the GAS7 F-BAR domain and the other F-BAR domains are indicated on the right. The deleted residues in the ΔFFL1 and ΔFFL2 mutants are indicated by the rectangles.

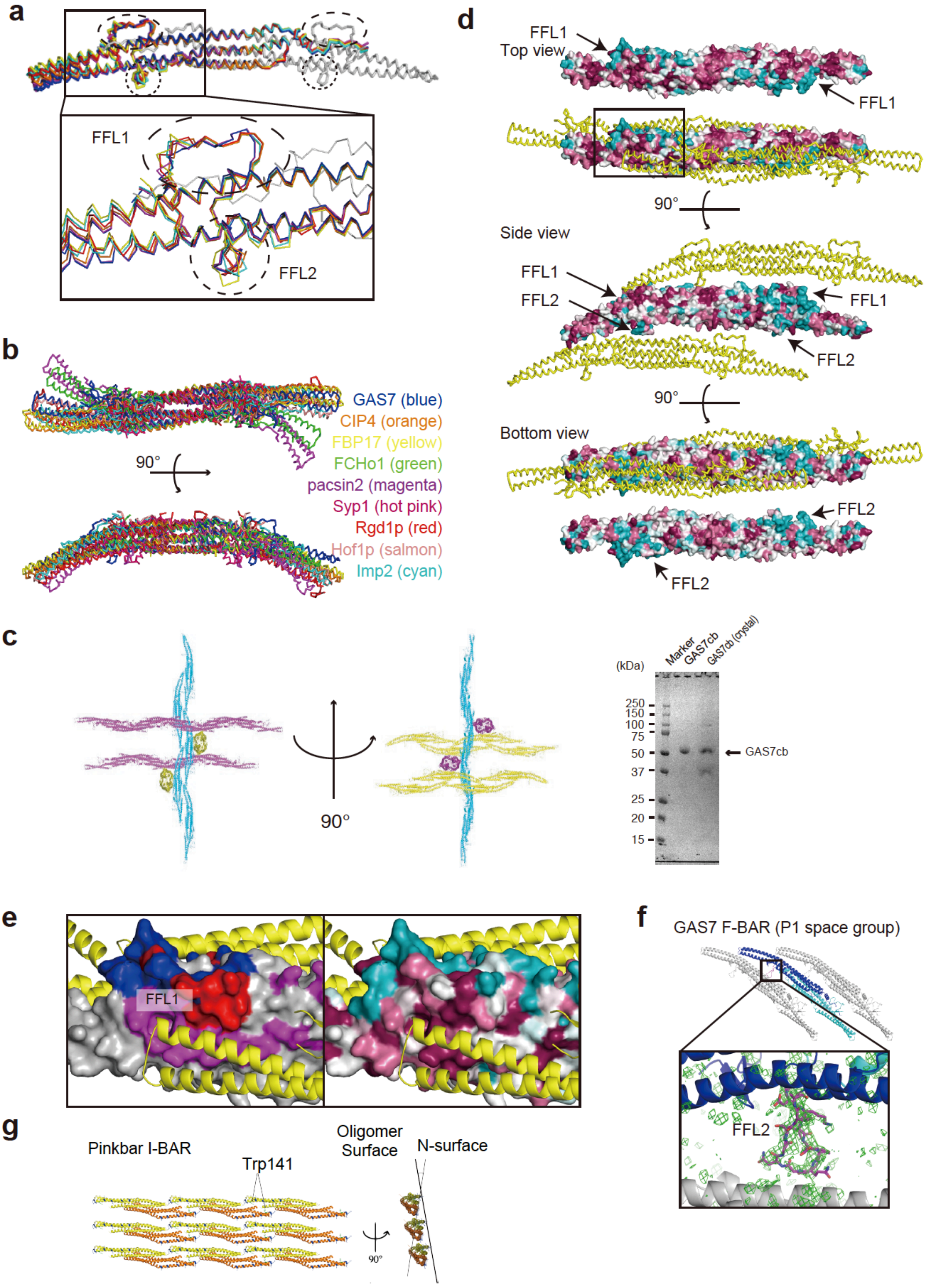
a
FFL1
FFL2
b
GAS7 (blue)
CIP4 (orange)
FBP17 (yellow)
FCHo1 (green)
pacsin2 (magenta)
Syp1 (hot pink)
Rgd1p (red)
Hof1p (salmon)
Imp2 (cyan)
90°
c
90°
(kDa)
Marker
GAS7cb
GAS7cb (crystal)
250
150
100
75
50
37
25
20
15
GAS7cb
d
FFL1
Top view
FFL1
90°
Side view
FFL1
FFL2
FFL1
FFL2
90°
Bottom view
FFL2
FFL2
e
FFL1
f
GAS7 F-BAR (P1 space group)
FFL2
g
Pinkbar I-BAR
Trp141
Oligomer Surface
N-surface
90°

Supplementary Figure 2. FFO in the crystal of the GAS7 F-BAR domain.

(**a**) Superimposition of the backbone Cα atom traces of the F-BAR domains in the asymmetric units of the crystals of the GAS7 F-BAR fragment (blue, cyan, magenta, and yellow) and GAS7cb (orange and red). One of the two molecules in the dimer is shown in grey. A close-up view of the FFLs is shown.

(**b**) Superimposition of the backbone Cα atom traces of the F-BAR dimers of GAS7 (blue), CIP4 (orange; PDB_ID: 2EFK), FBP17 (yellow; 2EFL), FCHo1 (green; 2V0O), pacsin2 (magenta; 3ABH), Syp1 (hot pink; 3G9G), Rgd1p (red; 4WPC), Hof1p (salmon; 4WPE), and Imp2 (cyan; 5C1F).

(**c**) Packing in the GAS7cb crystal. (left) The WW and SH3 domains of GAS7cb are not clearly visible in the electron density maps. Each filamentous GAS7 oligomer in the crystal packing is coloured differently. (right) The SDS-PAGE gel of the purified GAS7cb and the GAS7cb in the crystal.

(**d**) The surface of the GAS7 F-BAR dimer, coloured according to the degree of sequence conservation among the 46 close homologs in a gradient from cyan (most variable) to white to magenta (most conserved). The adjacent F-BAR dimers in the FFO are coloured yellow. The rectangle indicates the region magnified in (e).

(**e**) Close-up view of the interfaces between FFL1 and adjacent dimers in the FFO, indicated by the rectangle in (d). (Left) The amino-acid residues in the N-terminal extension before helix α1 are coloured blue (no binding to the adjacent dimer) or red (binding to the adjacent dimer). Other interface residues between the F-BAR dimers in the FFO are coloured magenta. (Right) The surface of the GAS7 F-BAR dimer is coloured by the sequence conservation, as in (d).

(**f**) Close-up view of the interaction between FFL2 and the adjacent F-BAR dimer in the FFO, with a simulated-annealing F_o-F_c omit electron density map contoured at +2.5σ. To create the map, all four FFLs in the asymmetric unit were omitted from the model.

(**g**) Comparison of GAS7 F-BAR and Pinkbar I-BAR in the crystals. Their membrane-binding surfaces and N-surfaces are shown. Trp141 of Pinkbar at the contacts between dimers in the crystals is indicated.

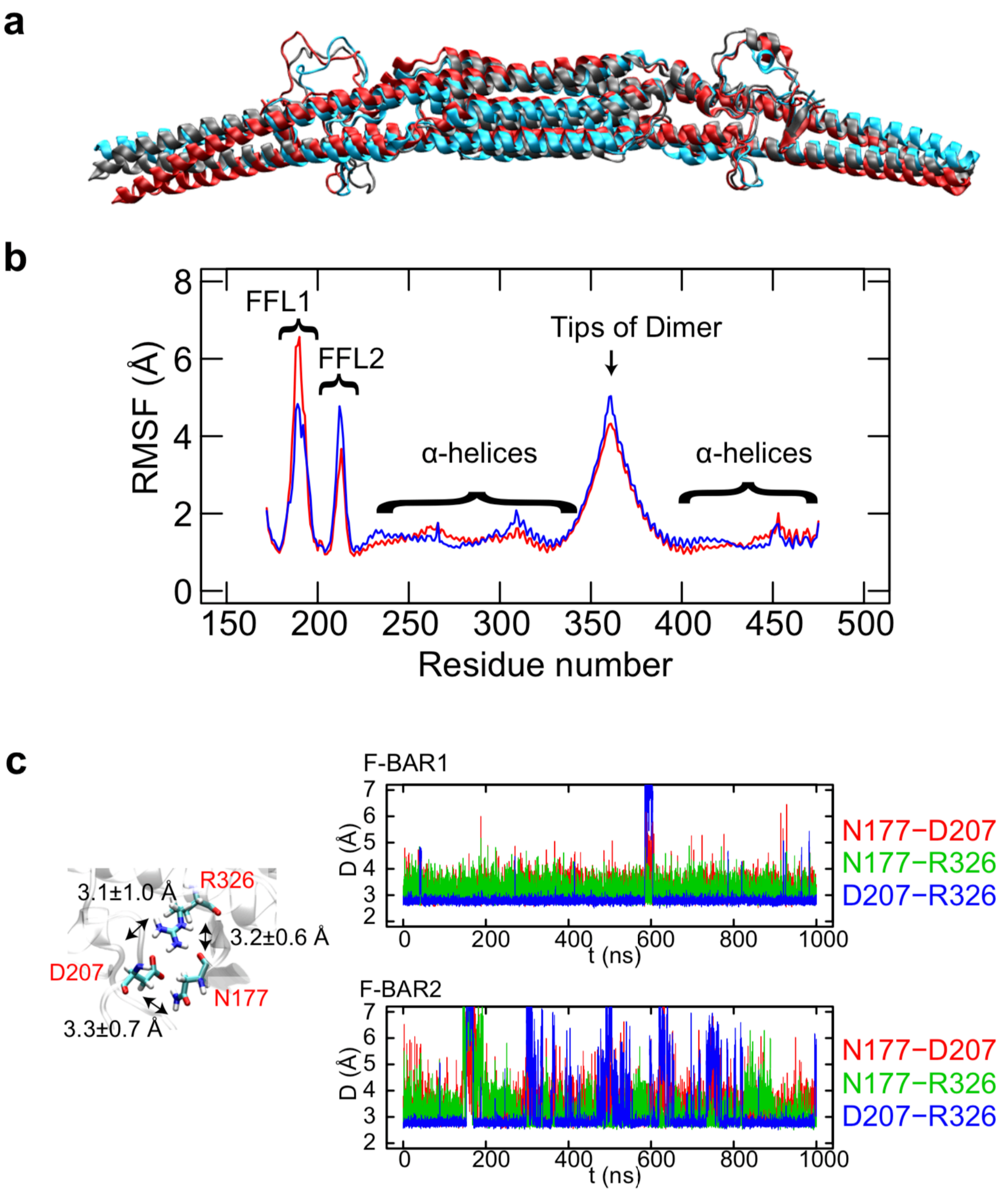

Supplementary Figure 3. Fluctuation of the GAS7 F-BAR domain during MD simulations.

(**a**) The average (black), the most highly curved (red), and the flattest (cyan) arrangement of F-BAR dimers in the MD simulation.

(**b**) Root mean square fluctuation (RMSF) of C_α atoms from the average structure, as a function of residue number.

(**c**) Averages and time evolutions of the distances between N177, D207, and R326. The mean ± S.D. are shown on the right.

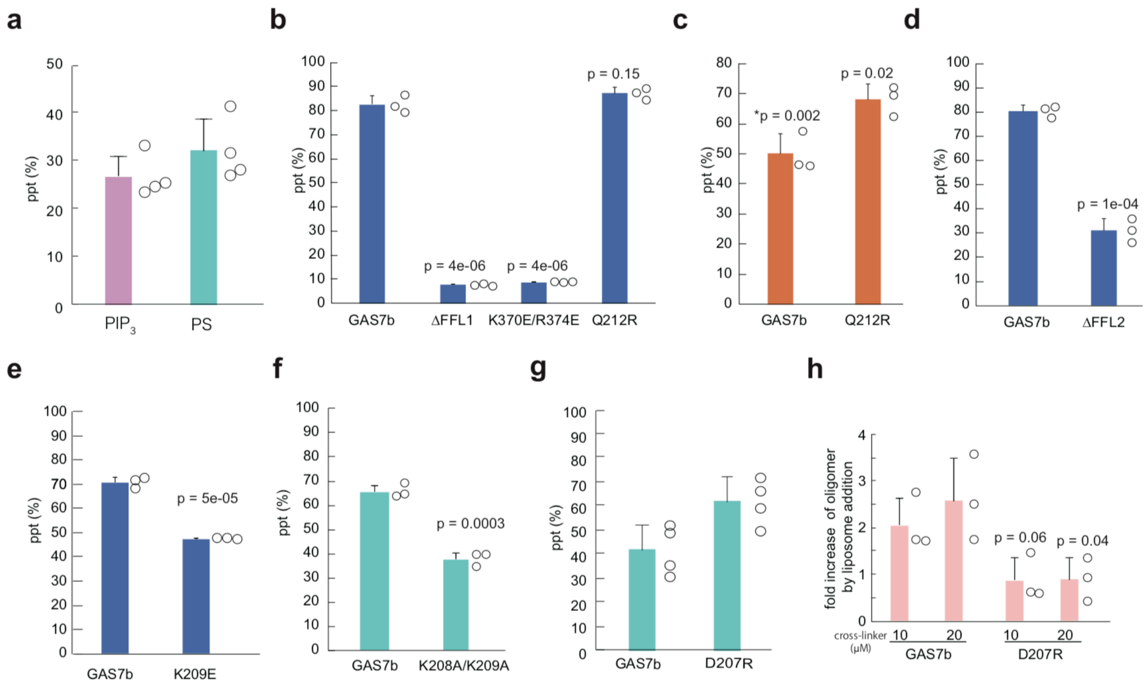

Supplementary Figure 4. Quantification of the proteins in the pellet in the liposome co-sedimentation assay.

Quantifications of the proteins in the pellets described in Figure 2 are shown. Source data are provided as a Source Data file.

(**a**) GAS7b (0.5 μM) to PS (PC:PE:PS=20:20:60) and PIP_3 (PC:PE:PS: PIP_3 =40:40:20:5) liposomes (0.2 mg ml^{-1}) (Figure 2f).

(**b**) GAS7b (wild type: WT) and its ΔFFL1, K370E/R374E, and Q212R mutants (0.5 μM) to bovine brain Folch liposomes (0.4 mg ml^{-1}) (Figure 2g).

(**c**) GAS7b and Q212R mutant (0.5 μM) to bovine brain Folch liposomes (0.4 mg ml^{-1}) in high salt buffer (Figure 2h).

(**d**) GAS7b and ΔFFL2 mutant (0.5 μM) to bovine brain Folch liposomes (0.4 mg ml^{-1}) (Figure 2k).

(**e**) GAS7b and K209E mutant (0.5 μM) to bovine brain Folch liposomes (0.4 mg ml^{-1}) (Figure 2i).

(**f**) GAS7b and K208A/K209A mutant (0.5 μM) to PS (PC:PE:PS=20:20:60) liposomes (0.4 mg ml^{-1}) (Figure 2j).

(**g**) GAS7b and D207R mutant (0.5 μM) to PS (PC:PE:PS=20:20:60) liposomes (0.2 mg ml^{-1}) (Figure 2l).

P-values were obtained using two-tailed Student's t-test relative to GAS7b (WT). P-

values (*) of the difference in the WT binding to liposomes in 200 mM NaCl (**b**) to that in high salt (300 mM NaCl) (**c**) buffers is shown in (**c**). The values of individual data are shown by circles. n=3-4. Error bars: S.D.

(**h**) Fold increase of the oligomer signals by the chemical cross-linking of GAS7b and of D207R mutant upon addition of PS (PC:PE:PS=20:20:60) liposomes (Figure 2m). Error bars show s.d..

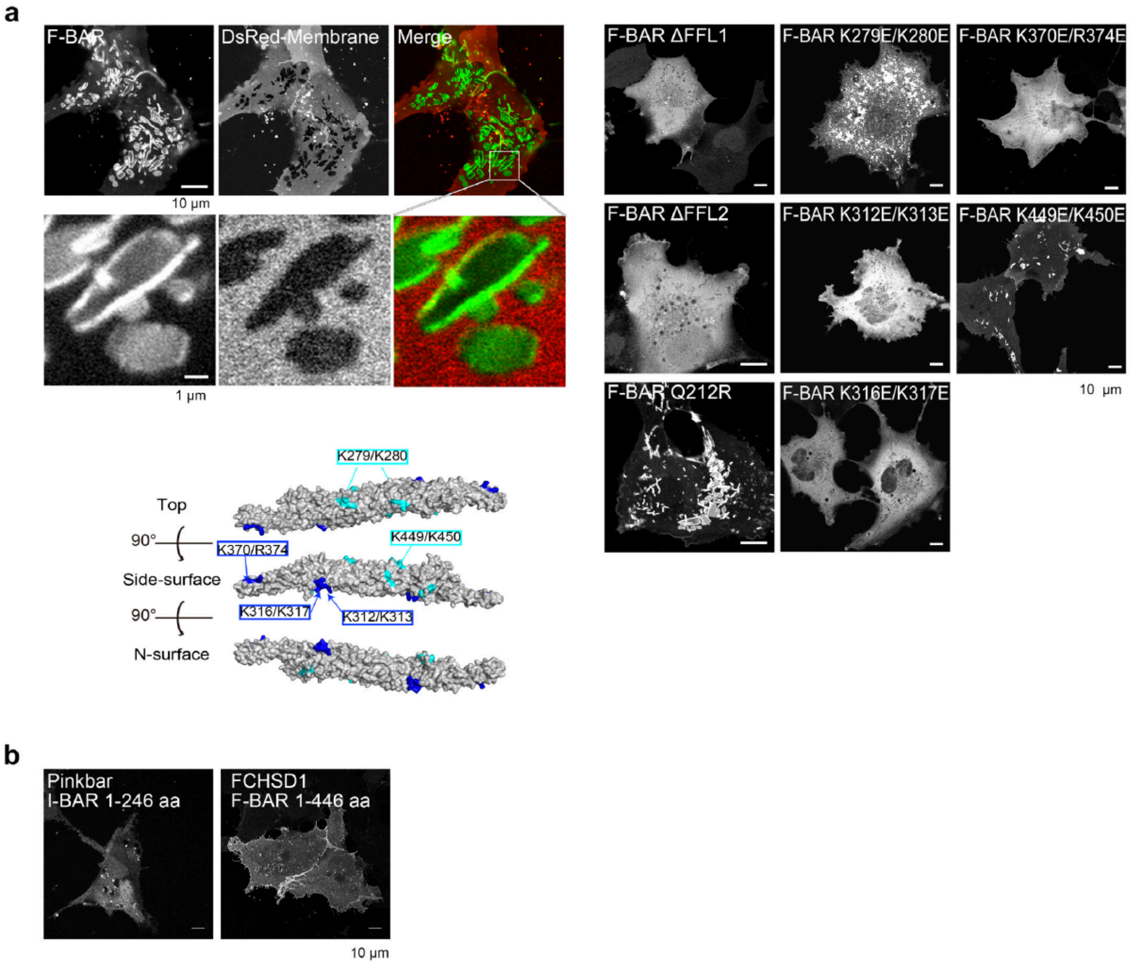

Supplementary Figure 5. Localizations of the F-BAR domain fragment of GAS7 and FCHSD1 and the I-BAR domain of Pinkbar in HeLa cells.

(**a**) GFP-GAS7 F-BAR domain and its ΔFFL1, ΔFFL2, Q212R, K279E/K280E, K312E/K313E, K316E/K317E, K370E/R374E, and K449E/K450E mutants (green for WT) expressed in HeLa cells. The plasma membranes of the cells were visualized by the co-expression of DsRed-membrane in F-BAR expressing cells (red). Scale bars: 10 μm. The K or R mutations with defects in the F-BAR domain assembly are shown in blue, while those without defects are shown in cyan on the F-BAR domain structure (left bottom). The enlargement of the WT is included to show the shape of the F-BAR assembly.

(**b**) GFP-Pinkbar I-BAR domain and GFP-FCHSD1 F-BAR domain expressed in HeLa cells. Scale bars: 10 μm.

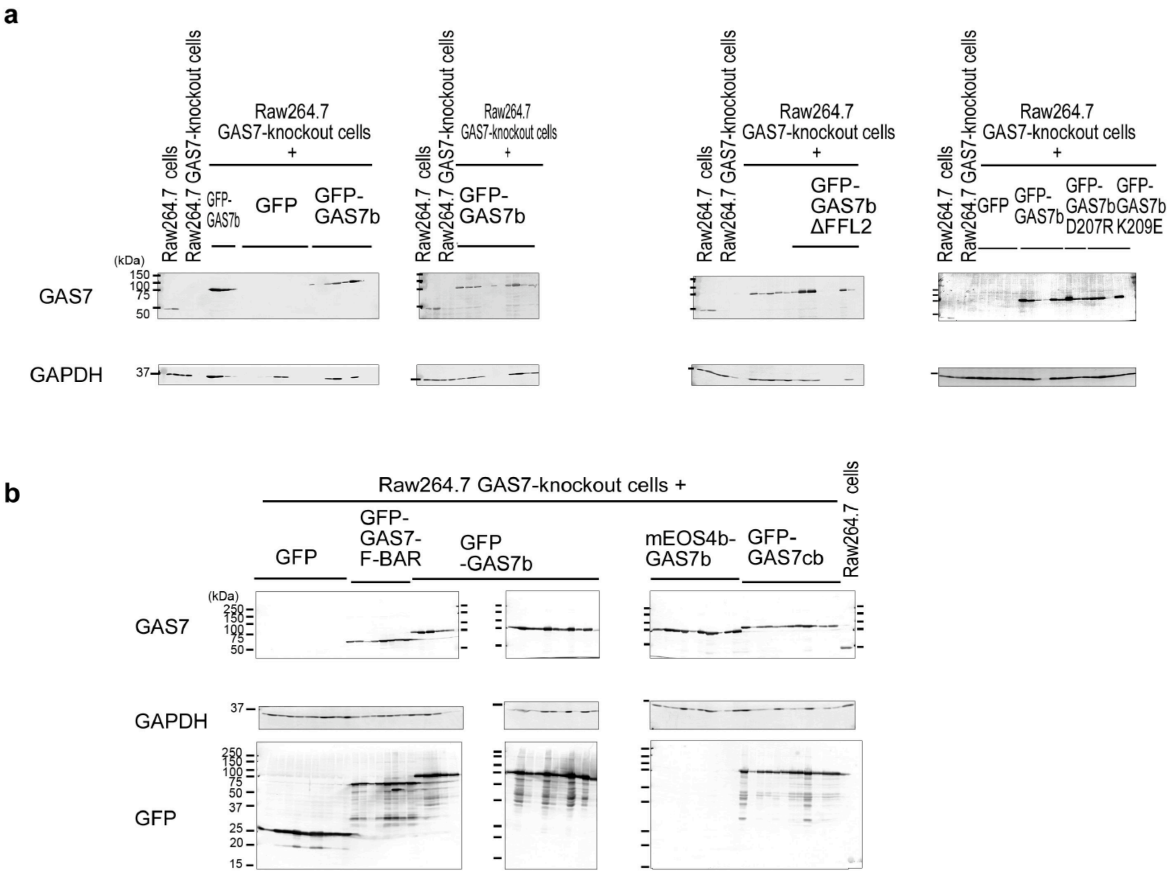

Supplementary Figure 6. Expression levels of GAS7 in macrophage clones.

Expression levels of GFP, GFP-GAS7b, GFP-GAS7cb, GFP-GAS7b mutants, and mEOS4b-GAS7b in GAS7-knockout RAW264.7 macrophage clones, as examined by western blotting using an anti-GAS7 antibody. GAS7-knockout cells were infected with the retrovirus expressing the indicated GAS7 constructs, and the cells were cloned. The amounts of GAPDH were examined as loading controls. GFP was also blotted using an anti-GFP antibody. (**a**) Clones for Fig. 3h-j. (**b**) Clones for Fig. 4 and Supplementary Fig. 7i.

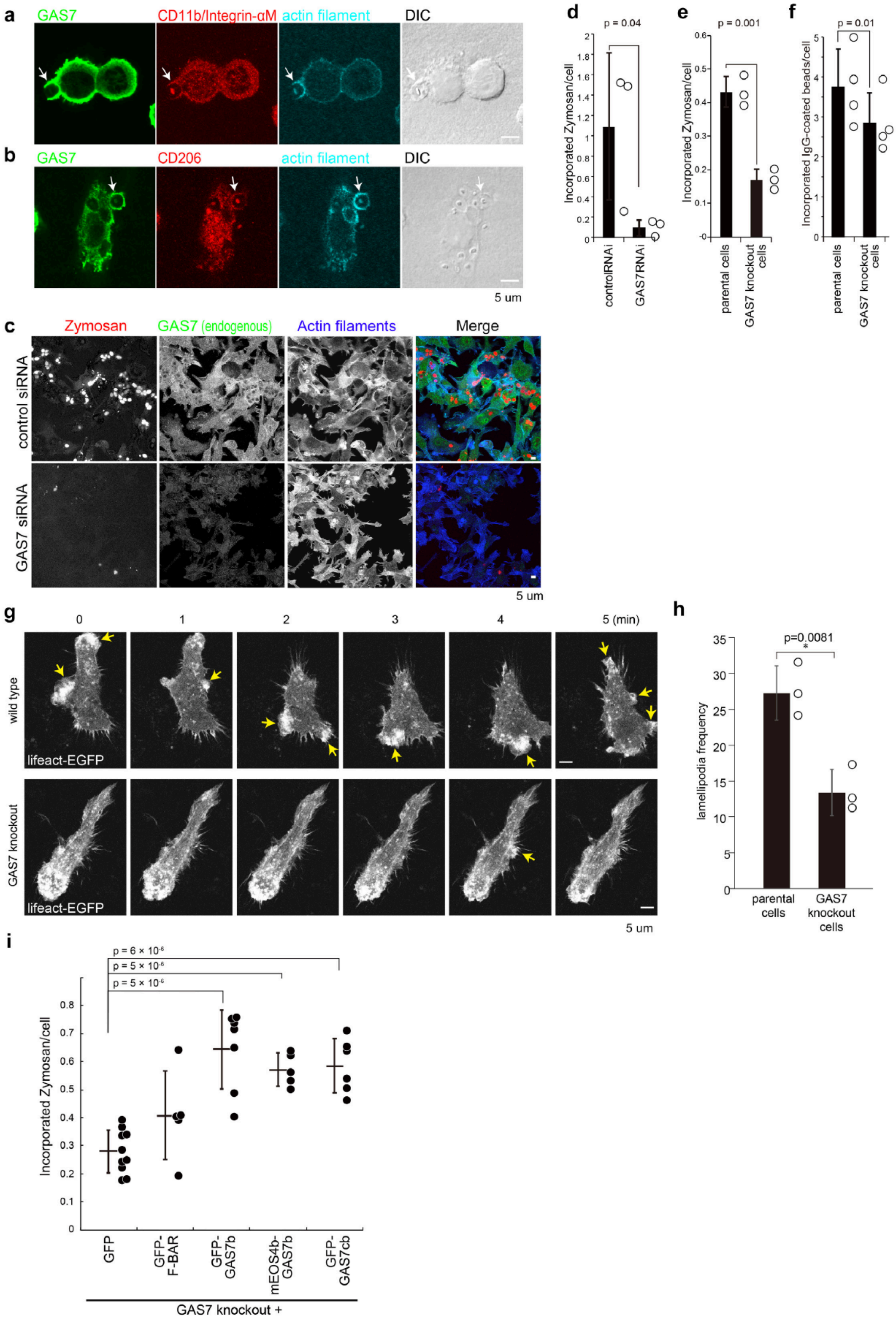
a
GAS7
CD11b/Integrin-αM
actin filament
DIC
b
GAS7
CD206
actin filament
DIC
5 um
c
Zymosan
GAS7 (endogenous)
Actin filaments
Merge
control siRNA
GAS7 siRNA
5 um
d
p = 0.04
Incorporated Zymosan/cell
controlRNAi
GAS7RNAi
e
p = 0.001
Incorporated Zymosan/cell
parental cells
GAS7 knockout cells
f
p = 0.01
Incorporated IgG-coated beads/cell
parental cells
GAS7 knockout cells
g
0
1
2
3
4
5 (min)
wild type
lifeact-EGFP
GAS7 knockout
lifeact-EGFP
5 um
h
p=0.0081
lamellipodia frequency
parental cells
GAS7 knockout cells
i
p = 6 × 10-6
p = 5 × 10-6
p = 5 × 10-6
Incorporated Zymosan/cell
GFP
GFP-F-BAR
GFP-GAS7b
mEOS4b-GAS7b
GFP-GAS7cb
GAS7 knockout +

Supplementary Figure 7. Phagocytosis mediated by GAS7 in macrophages.

(**a, b**) CD11b/integrin αM (complement receptor 3, α chain) (**a**) and CD206 (macrophage mannose receptor) (**b**) localization with GFP-GAS7b (green) expressed in GAS7-knockout RAW264.7 macrophages incorporating zymosan (DIC, arrow). Scale bars: 5 μm.

(**c**) RAW264.7 macrophages were treated with control or GAS7 siRNAs and then incubated with zymosan (red). GAS7 and actin filaments were visualized using an anti-GAS7 antibody (green) and phalloidin (blue) staining, respectively. Scale bars: 5 μm.

(**d**) Zymosan incorporation in cells treated with control siRNA or GAS7 siRNA. n=3. Error bars: S.D. P-values determined by the one-tailed Student's t-test relative to control cells are shown. The values of individual data are shown by circles.

(**e**) Zymosan incorporation in parental RAW264.7 cells or CRISPR/Cas9-mediated GAS7-knockout RAW264.7 cells. n=3. Error bars: S.D. P-values determined by the two-tailed Student's t-test relative to the parental cells are shown. The values of individual data are shown by circles.

(**f**) Incorporation of IgG-coated latex beads in RAW264.7 macrophages or the CRISPR/Cas9-mediated GAS7-knockout cells. n=4. Error bars: S.D. P-values determined by the paired two-tailed Student's t-test relative to control cells are shown. The values of individual data points are shown by circles.

(**g**) Time-lapse images of F-actin in RAW264.7 macrophage cells. Wild-type or GAS7-KO cells transiently expressing Lifeact-EGFP were subjected to live imaging at 1-min intervals. Representative still images are shown, and lamellipodia-like protrusions are indicated by arrows. Scale bar: 5 μm.

(**h**) Quantification of the frequency of lamellipodia formation over 15 min in wild-type or GAS7-knockout cells, using 7-15 cells per experiment. The graph represents the mean of three experiments. Error bars: S.D.

(**i**) Zymosan incorporation in GAS7b-knockout RAW264.7 macrophages expressing GFP, GFP-F-BAR, GFP-GAS7b, mEOS4b-GAS7b, or GFP-GAS7cb. Dots represent the average zymosan incorporation by each clone. GFP alone was expressed as a control. P-values determined by the two-tailed Student's t-test relative to GFP-expressing GAS7-knockout cells are shown.

Source data for d, e, f, h, and i are provided as a Source Data file. Error bars show s.d..

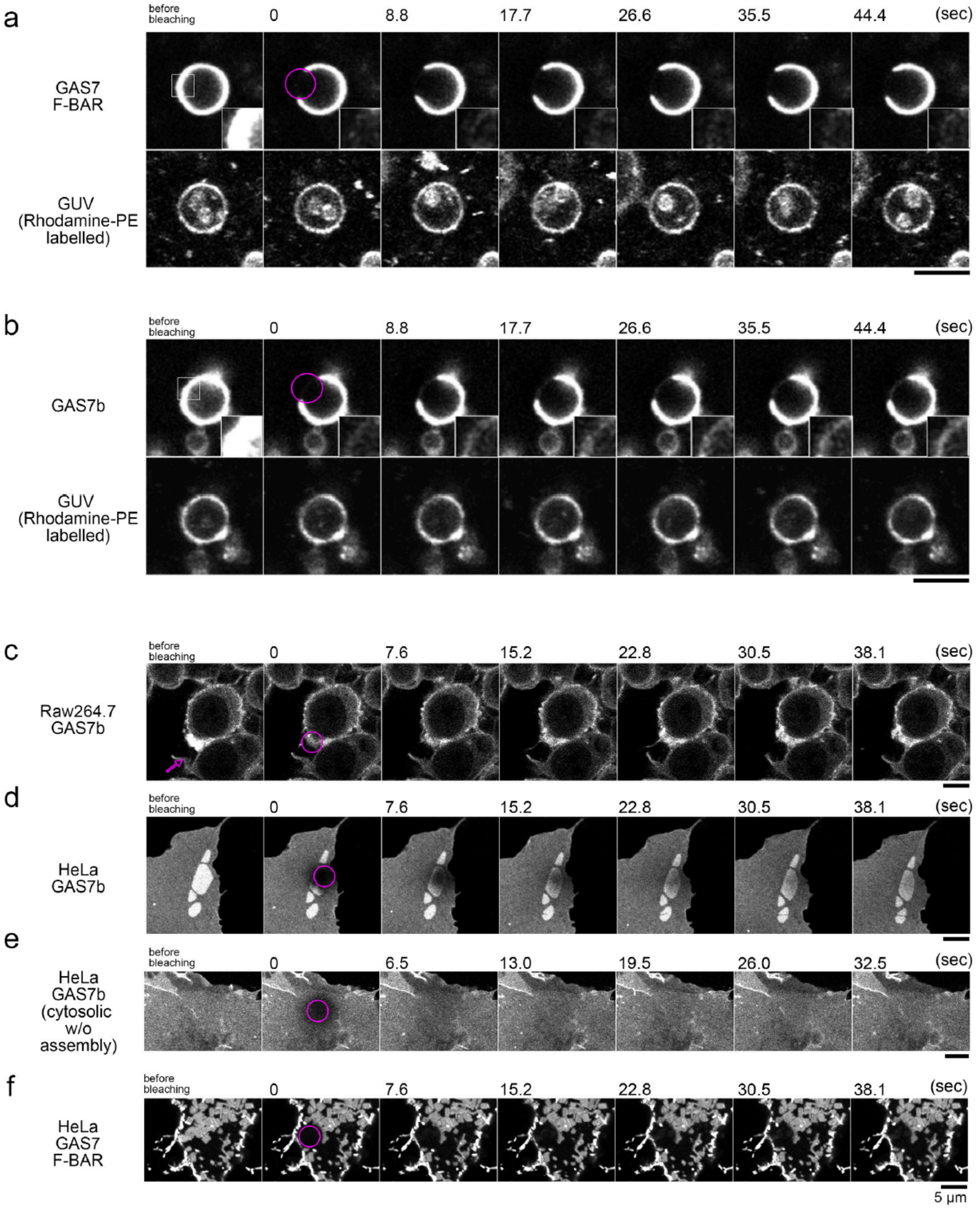

Supplementary Figure 8. Time-lapse images of fluorescence recovery after photobleaching.

Time-lapse images of fluorescence recovery after photobleaching of GFP-GAS7 F-BAR (**a**) and GFP-GAS7b (**b**) on GUVs, GFP-GAS7b (**c**) in RAW264.7 macrophages, and the assembled or cytosolic GFP-GAS7b (**d, e**) or the assembled GFP-GAS7 F-BAR (**f**)

in HeLa cells, related to Figure 3k, l. Magenta circles indicate the photobleached regions. The magenta arrow indicates the location of the zymosan particle. In (a, b), the Rhodamine-PE indicates the monolayered membranes, and the slight recovery of GFP-GAS7b on the membrane is shown in the enlarged images enclosed with squares. Scale bar: 5 μm.

Supplementary Table 1 Data collection, phasing, and refinement statistics

	GAS7 F-BAR domain (SeMet) (PDB ID 6IKN)	GAS7cb (Native) (PDB ID 6IKO)
**Data collection**		
Space group	*P*1	$P2_13$
Cell dimensions		
a, *b*, *c* (Å)	53.3, 87.3, 88.8	194.2, 194.2, 194.2
α, *β*, *γ* (°)	81.0, 74.7, 79.7	90.0, 90.0, 90.0
	Peak	
Wavelength (Å)	0.97890	0.90000
Resolution (Å)	50.00–3.00 (3.05–3.00)[a]	50.00–3.75 (3.81–3.75)
R_{merge}	0.283 (>1)	0.131 (>1)
$I/\sigma(I)$	7.0 (1.6)	22.4 (1.4)
$CC_{1/2}$[b]	0.97 (0.49)	0.99 (0.56)
Completeness (%)	99.1 (98.6)	99.9 (100.0)
Redundancy	6.4 (4.9)	20.8 (22.4)
**Refinement**		
Resolution (Å)	50.00–3.00	50.00–3.75
No. reflections	30,402	25,155
R_{work} / R_{free}	0.194/0.255	0.173/0.205
No. protein atoms	10,017	4,987
Average protein *B* factor (Å^2)	73.1	155.6
R.M.S. deviations		
Bond lengths (Å)	0.004	0.012
Bond angles (°)	0.94	1.57

Each structure was determined from a single crystal.

[a] Values in parentheses are for highest-resolution shell.

[b] Values calculated using all of the data.

Supplementary Table 2 Primers used in this study

Construct name (GAS7cb amino-acid residue numbers)	notes	Forward /Reverse	Primer Sequences for amplifying cDNA (5'-3')
pCold2 GAS7cb 1-476 aa mouse	Subcloned into Bam HI / Sal I sites of the vector after Bgl II /Sal I digestion.	Forward	GAGAAGATCTATGGCCGGCGCCCGCT
		Reverse	GAGAGTCGACCTAAATCTCCATGTCCACTGGGCGGAT
pCold2 GAS7 F-BAR 166-476 aa mouse	Subcloned into Bam HI / Sal I sites of the vector after Bam HI /Sal I digestion.	Forward	GAGAGGATCCAGGAAACAGAGCAAGGAAAACACC
		Reverse	GAGAGTCGACCTAAATCTCCATGTCCACTGGGCGGAT
pEGFP-C1- GAS7cb 1-476 mouse	Subcloned into Bgl II / Sal I sites of the vector after Bgl II /Sal I digestion.	Forward	GAGAAGATCTATGGCCGGCGCCCGCT
		Reverse	GAGAGTCGACCTAAATCTCCATGTCCACTGGGCGGAT
pEGFP-C1- GAS7F-BAR 166-476 mouse	Subcloned into Bgl II / Sal I sites of the vector after Bam HI /Sal I digestion.	Forward	GAGAGGATCCAGGAAACAGAGCAAGGAAAACACC
		Reverse	GAGAGTCGACCTAAATCTCCATGTCCACTGGGCGGAT
pEGFP-C1- GAS7b 62-476 mouse	pEGFP-C1- GAS7cb 1-476 was truncated to make GAS7b by site directed mutagenesis	Forward	ctgtacaagtccggactcagatctATGAAGCCTGGGATGGTCCCCC
		Reverse	GGGGGACCATCCCAGGCTTCATagatctgagtccggacttgtacag
pGEX6P-1 EGFP-GAS7cb 1-476 aa mouse	EGFP-GAS7b was transferred to pGEX6P-1 vector	Forward	GCCCCTGGGATCCCCGGTGGTGAGCAAGGGCGAGGAGCTGTTCAC
		Reverse	CGCTCGAGTCGACCCGGGTACAAATGTGGTATGGCTGATTATGA
pGEX6P-1 EGFP-GAS7b 62-476 aa mouse	pGEX6P-1 EGFP-GAS7cb 1-476 was truncated by site directed mutagenesis	Forward	ACCatggtcccccctccgccggg
		Reverse	cctggcggaggggggaccatGGTtctgagtccggacttgtaca
pGEX6P-1 mEOS4b-GAS7b 62-476 aa mouse	mEOS4b was amplified and EGFP was replaced.	Forward	TCCAGGGGCCCCTGGGATCCatggtgagtgcgattaagcc
		Reverse	cctggcggaggggggaccatGGTTCTGAGTCCGGAtcgtctgg
pGEX6P-1 GAS7cb 1-476 aa mouse	pGEX6P-1 EGFP-GAS7cb 1-476 was truncated by site directed mutagenesis	Forward	TCCAGGGGCCCCTGGGATCCatggccggcgcccgctgccg
		Reverse	GGATCCCAGGGGCCCCTGGAACAGAACTTC
pGEX6P-1 GAS7b 62-476 aa mouse	pGEX6P-1 EGFP-GAS7cb 1-476 was truncated by site directed mutagenesis	Forward	TCCAGGGGCCCCTGGGATCCatggtcccccctccaccagg
		Reverse	GGATCCCAGGGGCCCCTGGAACAGAACTTC
pGEX6P-1 GAS7 F-BAR 166-476 aa mouse	pGEX6P-1 EGFP-GAS7cb 1-476 was truncated by site directed mutagenesis	Forward	TCCAGGGGCCCCTGGGATCCaggaaacagagcaaggaaaac
		Reverse	GGATCCCAGGGGCCCCTGGAACAGAACTTC
pMXs-EGFP- GAS7cb 1-476 mouse	The EGFP-GAS7cb was transferred to pMXs vector	Forward	TACAAAAAAGCAGGCTCCGCgctagcgctaccggtcgccaccatg
		Reverse	TCGTCGACCACTGTGCTGGCcctctacaaatgtggtatggctgattatg
pMXs-EGFP- GAS7F-	pMXs-EGFP- GAS7cb 1-476 was	Forward	ctgtacaagtccggactcagatctATGAACCTGGGATCCTCATCGCC

BAR 157-476 mouse	truncated to make F-BAR by site directed mutagenesis	Reverse	GGCGATGAGGATCCCAGGTTCATagatctgagtccggacttgtacag
pMXs-EGFP- GAS7b 62-476 mouse	pMXs-EGFP- GAS7cb 1-476 was truncated to make GAS7b by site directed mutagenesis	Forward	ctgtacaagtccggactcagatctATGAAGCCTGGGATGGTCCCCC
		Reverse	GGGGGACCATCCCAGGCTTCATagatctgagtccggacttgtacag
GAS7 F-BAR ΔFFL1 (Δ171-197)	primers for site directed mutagenesis	Forward	GATCCAGGAAACAGAGCAAGTGGAGCTACTGTGACTACTT
		Reverse	AAGTAGTCACAGTAGCTCCACTTGCTCTGTTTCCTGGATC
GAS7b ΔFFL1 (Δ171-197)	primers for site directed mutagenesis	Forward	CAGGCAGGAAACAGAGCAAGTGGAGCTACTGTGACTACTT
		Reverse	AAGTAGTCACAGTAGCTCCACTTGCTCTGTTTCCTGCCTG
GAS7 ΔFFL2 (Δ209-216)	primers for site directed mutagenesis	Forward	ACTACTTTTGGGCGGACAAGGTGGCTGGCTTTGAACTGCT
		Reverse	AGCAGTTCAAAGCCAGCCACCTTGTCCGCCCAAAAGTAGT
GAS7 K208A/K209A	primers for site directed mutagenesis	Forward	CTGTGACTACTTTTGGGCGGACGCGGCGGACCCACAAGGCAATGG CACGG
		Reverse	CCGTGCCATTGCCTTGTGGGTCCGCCGCGTCCGCCCAAAAGTAGT CACAG
GAS7 K209E	primers for site directed mutagenesis	Forward	ACTTTTGGGCGGACAAGGAAGACCCACAAGGCAATGGCAC
		Reverse	GTGCCATTGCCTTGTGGGTCTTCCTTGTCCGCCCAAAAGT
GAS7 D207R	primers for site directed mutagenesis	Forward	GTGACTACTTTTGGGCGCGCAAGAAGGACCCACAAGGC
		Reverse	GCCTTGTGGGTCCTTCTTGCGCGCCCAAAAGTAGTCAC
GAS7 Q212R	primers for site directed mutagenesis	Forward	CGGACAAGAAGGACCCACGGGGCAATGGCACGGTGGCTGG
		Reverse	CCAGCCACCGTGCCATTGCCCCGTGGGTCCTTCTTGTCCG
GAS7 K370E/R374E	primers for site directed mutagenesis	Forward	GGACATCGAGAAGGCACGGGAGAAGTCCACTCAGGCCGGAGATG
		Reverse	GCCTGAGTGGACTTCTCCCGTGCCTTCTCGATGTCCTCCTCGGTC
pCAG-EGxxFP GAS7	Amplification of the mouse genome for reporter of pX330-GAS7 transfection.	Forward	GCTGCCCGACAACCACtgaggatccACAGACCTCAGCGGGTATAG
		Reverse	GGTCAGCTTGCCgatatcgaattcCAACCAGAGGTGGAAATGGAGG
pX330-GAS7	Oligos were annealed and ligated into BbsI digested vector.	Forward	caccGGCGGAGGGGGACCATCCC
		Reverse	aaacGGGATGGTCCCCCTCCGCC
