# Supplementary material for: Phagocytosis is mediated by two-dimensional assemblies of the F-BAR protein GAS7
Source: Nat Commun. 2019 Oct 18;10:4763. doi: 10.1038/s41467-019-12738-w (PMC6802115; doi:10.1038/s41467-019-12738-w)
Supplement: Supplementary file 3 — Reporting Summary [file 41467_2019_12738_MOESM3_ESM.pdf]

nature research

Corresponding author(s): Shiro Suetsugu, Atsushi Shimada

☐ Initial submission ☐ Revised version ☒ Final submission

Life Sciences Reporting Summary

Nature Research wishes to improve the reproducibility of the work that we publish. This form is intended for publication with all accepted life science papers and provides structure for consistency and transparency in reporting. Every life science submission will use this form; some list items might not apply to an individual manuscript, but all fields must be completed for clarity.

For further information on the points included in this form, see Reporting Life Sciences Research. For further information on Nature Research policies, including our data availability policy, see Authors & Referees and the Editorial Policy Checklist.

ü Experimental design

1. Sample size

Describe how sample size was determined.

Cell biological and biochemical experiments were conducted with more than triplicate replicates, thus allowing for statistical testing.

2. Data exclusions

Describe any data exclusions.

Not applicable to this study.

3. Replication

Describe whether the experimental findings were reliably reproduced.

Experiments were repeated with different samples and days. At least three experiments were replicated and gave similar results.

4. Randomization

Describe how samples/organisms/participants were allocated into experimental groups.

The fields for counting were randomly selected.

5. Blinding

Describe whether the investigators were blinded to group allocation during data collection and/or analysis.

Not applicable to this study because this study does not involve animals and human patients.

Note: all studies involving animals and/or human research participants must disclose whether blinding and randomization were used.

6. Statistical parameters

For all figures and tables that use statistical methods, confirm that the following items are present in relevant figure legends (or in the Methods section if additional space is needed).

n/a	Confirmed	
☐	☒	The exact sample size (*n*) for each experimental group/condition, given as a discrete number and unit of measurement (animals, litters, cultures, etc.)
☐	☒	A description of how samples were collected, noting whether measurements were taken from distinct samples or whether the same sample was measured repeatedly
☐	☒	A statement indicating how many times each experiment was replicated
☐	☒	The statistical test(s) used and whether they are one- or two-sided (note: only common tests should be described solely by name; more complex techniques should be described in the Methods section)
☐	☒	A description of any assumptions or corrections, such as an adjustment for multiple comparisons
☐	☒	The test results (e.g. *P* values) given as exact values whenever possible and with confidence intervals noted
☐	☒	A clear description of statistics including central tendency (e.g. median, mean) and variation (e.g. standard deviation, interquartile range)
☐	☒	Clearly defined error bars

See the web collection on statistics for biologists for further resources and guidance.

Software

Policy information about availability of computer code

7. Software

Describe the software used to analyze the data in this study.	See methods section for details. XDS, HKL200, PHENIX, autoSHARP, pymol, NIS-elements, ViSP, ThunderSTORM, Excel, Matlab.<br>The Matlab code will be put on GitHub upon publication.

For manuscripts utilizing custom algorithms or software that are central to the paper but not yet described in the published literature, software must be made available to editors and reviewers upon request. We strongly encourage code deposition in a community repository (e.g. GitHub). *Nature Methods* guidance for providing algorithms and software for publication provides further information on this topic.

Materials and reagents

Policy information about availability of materials

8. Materials availability

Indicate whether there are restrictions on availability of unique materials or if these materials are only available for distribution by a for-profit company.	No such reagents and materials were used in this study.

9. Antibodies

Describe the antibodies used and how they were validated for use in the system under study (i.e. assay and species).	Anti-GAS7 antibody, clone 2F6, mouse monoclonal, from Origene (cat. no.TA501756), was validated by the Western blotting using the lysates of control and GAS7 siRNA-treated RAW264.7 cells.<br>Anti-actin antibody, clone C4, mouse monoclonal, from Millipore (cat. no. MAB1501) was used for loading control and was validated by the Western blotting.<br>Anti-GFP antibody, rabbit polyclonal, from MBL international (cat. no. 598) was validated by the Western blotting using lysates of GFP transfected cells.<br>Anti-integrin-αM (Ox42) and anti-CD206 (Santa Cruz); rabbit anti-N-WASP (clone 30D10, Cell Signaling); mouse anti-Arp3 (clone FMS338, Abcam); rabbit anti-GFP (598, MBL); and rat anti-GFP (clone GF090R, Nacalai) antibodies were validated Western blotting using the lysate of RAW264.7 cells.

10. Eukaryotic cell lines

a. State the source of each eukaryotic cell line used.	The RAW264.7 cells were from Riken (RCB0535).<br>The PLAT-A cells were from Dr Toshio Kitamura at University of Tokyo.<br>The HeLa cells were from Tadaomi Takenawa Lab in Univ Tokyo, originally from ATCC.
b. Describe the method of cell line authentication used.	The authentication was done by the provider (http://cell.brc.riken.jp/en/quality). We do not find any discrepancy to the validation report or previous literatures.
c. Report whether the cell lines were tested for mycoplasma contamination.	The mycoplasma contamination was tested. We did not detect any sign of contamination.
d. If any of the cell lines used are listed in the database of commonly misidentified cell lines maintained by ICLAC, provide a scientific rationale for their use.	Not applicable to this study.

Animals and human research participants

Policy information about studies involving animals; when reporting animal research, follow the ARRIVE guidelines

11. Description of research animals

Provide details on animals and/or animal-derived materials used in the study.	Not applicable to this study.

Policy information about studies involving human research participants

12. Description of human research participants

Describe the covariate-relevant population characteristics of the human research participants.	Not applicable to this study.
